# Familial CD45RA^–^ T cells to treat severe refractory infections in immunocompromised patients

**DOI:** 10.3389/fmed.2023.1083215

**Published:** 2023-02-08

**Authors:** Karima Al-Akioui Sanz, Carlos Echecopar Parente, Cristina Ferreras, Marta Menéndez Ribes, Alfonso Navarro, Carmen Mestre, Laura Clares, José Luis Vicario, Antonio Balas, Raquel De Paz, Eduardo López Granados, Elena Sánchez Zapardiel, Carlos Jiménez, María López-Oliva, Esther Ramos, Francisco Hernández-Oliveros, Antonio Pérez-Martínez

**Affiliations:** ^1^Hospital La Paz Institute for Health Research, Madrid, Spain; ^2^Department of Pediatric Hemato-Oncology, La Paz University Hospital, Madrid, Spain; ^3^Histocompatibility Unit, Transfusion Center of Madrid, Madrid, Spain; ^4^Cell Therapy Unit, Department of Hematology, La Paz University Hospital, Madrid, Spain; ^5^Immunology Unit, La Paz University Hospital, Madrid, Spain; ^6^Department of Nephrology, La Paz University Hospital, Madrid, Spain; ^7^Intestinal Rehabilitation Unit, Pediatric Gastroenterology and Nutrition Unit, La Paz University Hospital, Madrid, Spain; ^8^Department of Pediatric Surgery, La Paz University Hospital, Madrid, Spain; ^9^Department of Pediatrics, Faculty of Medicine, Universidad Autónoma de Madrid, Madrid, Spain

**Keywords:** CD45RA^–^ T cells, adoptive cell therapy (ACT), BK virus (BKV), Epstein-Barr virus (EBV), Cytomegalovirus (HCMV), *Aspergillus*, mDLI

## Abstract

**Background:**

Immunocompromised patients are susceptible to high-risk opportunistic infections and malignant diseases. Most antiviral and antifungal drugs are quite toxic, relatively ineffective, and induce resistance in the long term. The transfer of pathogen-specific Cytotoxic T-Lymphocytes has shown a minimal toxicity profile and effectiveness in treating Cytomegalovirus, Adenovirus, Epstein - Barr virus, BK Virus and *Aspergillus* infections, but this therapy have the main limitations of regulatory issues, high cost, and absence of public cell banks. However, CD45RA^–^ cells containing pathogen-specific memory T-cells involve a less complex manufacturing and regulatory process and are cheaper, feasible, safe, and potentially effective.

**Methods:**

We present preliminary data from six immunocompromised patients: four who had severe infectious diseases and two who had EBV lymphoproliferative disease. All of them underwent multiple safe familial CD45RA^–^ T-cell infusions as adoptive passive cell therapy, containing Cytomegalovirus, Epstein - Barr virus, BK virus, and *Aspergillus*-specific memory T-cells. We also present the method for selecting the best donors for CD45RA^–^ cells in each case and the procedure to isolate and store these cells.

**Results:**

The infusions were safe, there was no case of graft-versus host disease, and they showed a clear clinical benefit. The patients treated for BK virus nephritis, Cytomegalovirus encephalitis, Cytomegalovirus reactivation, and disseminated invasive aspergillosis experienced pathogen clearance, complete resolution of symptoms in 4-6 weeks and a lymphocyte increase in 3 of 4 cases after 3–4 months. Donor T cell transient microchimerism was detected in one patient. The two patients treated for EBV lymphoproliferative disease underwent chemotherapy and several infusions of CD45RA^–^ memory T-cells containing EBV cytotoxic lymphocytes. Donor T-cell microchimerism was observed in both patients. The viremia cleared in one of the patients, and in the other, despite the viremia not clearing, hepatic lymphoproliferative disease remained stable and was ultimately cured with EBV-specific Cytotoxic T-Lymphocytes.

**Conclusion:**

The use of familial CD45RA^–^ T-cells containing specific Cytotoxic T-lymphocytes is a feasible, safe and potential effective approach for treating severe pathogen infections in immunocompromised patients through a third party donor. Furthermore, this approach might be of universal use with fewer institutional and regulatory barriers.

## 1. Introduction

Advances in supportive care continue to improve the outcomes of immunocompromised patients. However, despite the improvements in achieving early diagnosis, the use of prophylactic therapies and the establishment of early treatment, infectious diseases remain a significant cause of morbidity and mortality in patients with immuno-deficiencies. These immunocompromised patients are also susceptible to Epstein-Barr virus (EBV) infections or reactivations, responsible in many cases for the post-transplantation lymphoproliferative disease (PTLD) (1). These patients are more susceptible to infection due to lack of pathogen-specific T-cell immunity, which is essential for resolving several infectious diseases, especially those caused by viruses (2, 3).

Drug therapies involve costly agents, are associated with side effects and do not act on the main cause of the severity, latency and recurrence of the infection, which is the absence or evasion of specific cellular immunity (4). Cell therapies present a new paradigm in treating both infectious and tumor diseases. Between approved T-cell therapies we find Donor Lymphocyte Infusions (DLI) of Cytotoxic T-Lymphocytes (CTLs) to reestablish the immune system after a transplant or to treat Severe Combined Immunodeficiencies (5–9). The infusion of specific CTLs has also been shown effective and safe in treating refractory infections in immunocompromised patients, especially those infections caused by Human Cytomegalovirus (HCMV), Adenovirus (AdV), EBV and BK Virus (BKV) (4, 10, 11). For patients whose donor lacks virus-specific cellular immune memory, the use of third party CTLs has been shown to have a safe clinical effect and has been associated with long-term viral control (10, 12). For these patients, a higher Human Leukocyte Antigens (HLA)-match has been associated with better systemic survival of the transferred cells (13). However, the generation of CTLs is a costly process in both time and resources, requiring highly skilled manufacturing technologists and the regulation issues can be struggling (10, 14, 15).

A simpler and cheaper alternative to the use of CTLs is the mDLI (memory donor lymphocyte infusion) of CD45RA^–^ T cells. The CD45RA^+^ fraction is supposed to cause lethal graft-versus-host disease (GvHD), but the mDLI of CD45RA^+^ depleted cells has proven to be safe and effective, given that these cells are less alloreactive. That is because of their lower proportion of naïve-T cells and greater proportion of memory T-cells (5, 7, 15–18). CD45RA^–^ cells from a familiar donor or a third-party donor, which contains pathogen-specific memory T-cells, can be isolated from the peripheral blood of a convalescent donor and infused directly into the patient (6–8, 17, 19, 20).

Here we present a treatment focused on clearing virus and specific fungus by mDLIs of CD45RA^–^ cells containing pathogen-specific memory T-cells from a healthy donor. The objective is providing and improving the cellular immunity of the patient until he recovers its own one. This procedure might be effective in any T-cell mediated infection, above all when immunocompromised patients present sustained lymphopenia. However, we have tested it only with viruses HCMV, EBV, BKV, and AdV, and the fungus *Aspergillus*, which usually act as opportunistic or latent pathogens that cause problems in immunocompromised patients, and that are becoming increasingly resistant to anti-viral or anti-fungal drugs. With emergent viruses such as SARS-CoV-2, this procedure is being tested in a clinical trial with promising results (20), so we decided to extend this procedure to other infectious diseases. We also present the methodology to detect, isolate and infuse those CD45RA^–^ cells containing pathogen-specific memory T-cells in a novel cell therapy, with major potential for use in all hospitals in the near future.

## 2. Materials and methods

### 2.1. Patients characteristics and diseases

**FIGURE 1:**
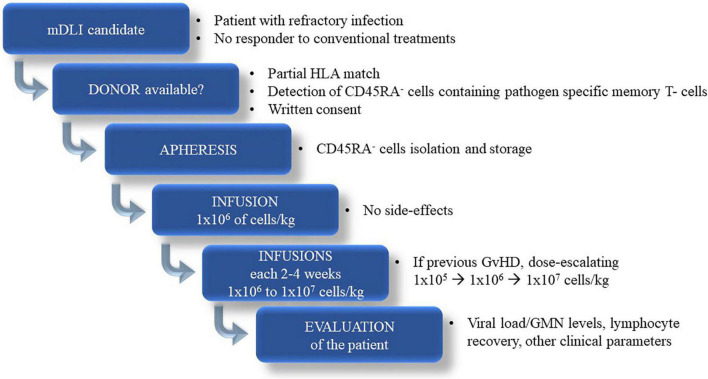
Scheme describing the general procedure followed in our center to treat immunocomprimised patients with infectious diseases.

We conducted in the University Hospital La Paz a study with immunosuppressed patients (five children and one adult) who suffered with refractory and severe infectious disease or lymphoproliferative disease associated with EBV that did not respond to classical management. Table 1 lists the patients’ characteristics. In all cases this therapy was applied when antivirals or antifungals ineffective and/or organ toxicity. A general scheme of the whole procedure is represented in Figure 1.

**TABLE 1:** Patient characteristics and pathogen-associated disease.

Patient	Sex	Age (years)	Primary disease	Immuno-suppression therapy	Lympho-cytes at time of infection	Infectious disease	Viral copies or GMN at diagnosis (copies/μl)	Standard therapy and duration	Viral copies or GMN after standard therapy (copies/μl)
1	F	37	Kidney transplantation	Prednisone Everolimus Tacrolimus	670/μl	BKV nephritis	5.6 × 10^5^	IS minimization, leflunomide and IGs (4 months)	7.5 × 10^3^
2	M	19	Chronic granulomatous disease and MUD HSCT	Methylprednisolone (0.5 mg/kg/d) Cyclosporin	50/μl	HCMV encephalitis	3.69 × 10^7^ in LCR, 4.97 × 10^3^ in serum	Foscarnet (7 days), ganciclovir and specific HCMV IGs (parenteral and intrathecal) (2 months)	2.3 × 10^3^
3	M	7	Multivisceral transplantation	Methylprednisolone (2 mg/kg/d) Sirolimus	1.310/μl	HCMV systemic infection	1.14 × 10^3^	Ganciclovir (2 months), Foscarnet (1 month) and weekly IGs (2.5 months)	< 1,000
4	F	15	CTLA4 haploinsufficiency and MUD HSCT	Methylprednisolone 0.4 mg/kg/day Abatacept	870/μl	Lung disseminated invasive aspergillosis	2.99	Micafungin. Surgical resection (x2). Voriconazole and amphotericin B (2 years).	0.52
5	F	12	Multivisceral transplantation	Methylprednisolone 0.3 mg/kg/day Tacrolimus Everolimus	230/μl	Liver EBV PTLD	1.2 × 10^5^	IS minimization, Rituximab and chemotherapy (2,5 years)	1.07 × 10^5^
6	F	9	Primary Immunodefficiency	None	340/μl	EBV DLBCL	4.09 × 10^5^	Rituximab and chemotherapy (8 months)	0

BKV, BK virus; HCMV, cytomegalovirus; DLBCL, diffuse large B-cell lymphoma; EBV, Epstein Barr virus; HSCT, hematopoietic stem cell transplantation; MUD, match unrelated donor; PTLD, post-transplant lymphoproliferative disease; GMN, galactomannan; IG, immunoglobulin; IS, immunosuppression.

### 2.2. Donor selection, cell processing, and detection of specific memory T-cells

The donors were family members in all cases, and the selection was based on HLA genotype compatibility with the patients after hematopoietic stem cell transplantation (HSCT) (it should be at least partial match) and the presence of specific memory T-cells against the pathogen related to the patients’ disease. These data is included in Table 2. After providing their informed consent, HLA typing of the eligible donors was performed at the Community of Madrid Transfusion Center (Madrid, Spain) on two independent samples by sequence-specific oligonucleotide and next-generation sequencing.

**TABLE 2:** Donor characteristics.

Patient	Familiar donor, specificity	IFN-γ^+^ T cells (%)	HLA-matching
1	Brother (Match)	0.4	A*24 B*44 B*45 C*02 C*16 DRB1*01 DRB1*10 DQB1*05
2	Brother (Mismatch related donor)	1.61	A*03, DRB1*07 DQB1*02
3	Mother (Haploidentical)	0.62	A*68 B*51 C*15 DRB1*08 DQB1*04
4	Father (Haploidentical)	0.28	A*11 C*07 B*49 DRB1*13 DQB1*06
5	Father (Haploidentical)	0.17	DRB1*07 DRB1*13 DRB1*02 DRB1*06
6	Mother (Haploidentical)	0.3	A*24 C*04 B*35 DRB1*11 DQB1*03 DPB1*04

The assay to check the presence of specific memory T-cells against a particular pathogen was performed as previously described by our group with certain modifications (19). Briefly, peripheral blood mononuclear cells (PBMCs) samples were collected from potential healthy donors and isolated using density gradient centrifugation (Ficoll-Paque; GE Healthcare, Chicago, IL, USA). Cells were resting overnight (O/N) in TexMACS medium (Miltenyi Biotec, Germany) supplemented with 10% AB serum (Sigma-Aldrich, Saint Louis, MO, USA) and 1% penicillin/streptomycin (Sigma-Aldrich). Approximately 1 million cells were then stimulated with specific peptides at a final concentration of 0.6 nmol/ml. Peptides are small fragments measuring 8–12 amino acids in length that cover specific epitopes of the virus. In this case the peptides used were PepTivator^®^ CMV pp65 (UniProt ID: P06725, Miltenyi Biotec) for HCMV; EBV-PT consensus premium grade human (Miltenyi Biotec) for EBV; BKV small T (ST) antigen (UniProt ID: P15000), BKV LT antigen (UniProtKB Acc. no. P14999), BKV VP1 capsid protein (UniProt ID: P14996) and BKV VP2 protein (UniProt ID: P14997) for BKV (Miltenyi Biotec); and PepTivator^®^ AdV Select for AdV, a peptide pool of different proteins coming from virus serotypes 2 and 5 (Miltenyi Biotec). For *Aspergillus fumigatus* we used the MACS GMP *Aspergillus fumigatus* Lysate 4 mg, (Miltenyi Biotec), with a final concentration of 1 μg/ml.

To detect specific memory T- cells against BKV, AdV, or *Aspergillus*, cells were rested O/N at 37°C in supplemented medium After 5 h stimulation with pooled peptivators or the *Aspergillus* lysate, depending on each case, interferon-gamma (IFN-γ) was caught and labeled (Human IFN-γ Secretion Assay-Detection Kit, Miltenyi Biotec). Basal IFN-γ production by PBMCs was included as a background control in the absence of stimulation. To consider a sample positive it had to have 0.1% of IFN-γ^+^ cells (0.01% for EBV) out of the total cells with a minimum of 150,000 events analyzed. In addition, the sample had to contain at least twice the number of IFN-γ^+^ cells than in the negative control, as well as positive control based on plate-bound cells stimulated with mouse anti-human CD3 and co-stimulated with purified CD28/CD49d. After incubation, cells where stained using the following fluorochrome-conjugated anti-human surface antibodies: CD45RA FITC, CD27 APC, CD3 VioGreen, CD4 PECy7, CD8 APC Cy7, L/D 7AAD, and IFN-γ PE. Cell acquisition was then performed using a Navios cytometer (Beckman Coulter), acquiring a mean of 150,000 cells. To detect specific memory T- cells against CMV and EBV, same process was performed, but incubation was for 6 h and adding Brefeldin A (Sigma-Aldrich), and IFN-γ production capacity was analyzed by intracellular flow cytometry staining. The antibodies used were the following: surface antibodies 7-AAD PC5.5, CD45 KO, CD8 APC-AF700, and CD4 APC-AF750 (Beckman Coulter Inc.), and intracellular marker IFN-γ- PacificBlue (Beckman Coulter). The cell adquisition was made by DxFlex cytometer (Beckman Coulter). The analysis was performed using FlowJo 10.7.1 software (FlowJo LLC) in all cases.

### 2.3. Donor inclusion and CD45RA T-cell depletion

After confirmation of HLA compatibility and a positive memory T-cells response against the pathogen of interest, the donors’ clinical history was reviewed and a physical examination was done. Non-mobilized apheresis was then performed at the Bone Marrow Transplantation and Cell Therapy Unit of University Hospital La Paz (Madrid, Spain) using a CliniMACS Plus cell separation system (Miltenyi Biotec).

### 2.4. CD45RA^–^ T cells infusion

Obtained allogenic CD45RA^–^ T cells were frozen as previously described (20) and infused into the patients weekly and then monthly depending on the cases and the patients’ state. Table 3 includes number of cell infusions and cells dose administrated in each case.

**TABLE 3:** Infusion characteristics, response and outcomes after therapy with familial CD45RA^–^ T cells containing pathogen-specific memory T cells (BAL, Broncho-alveolar lavage; GMN, galactomannan).

Patient	Number of infusions	Dose of infusions (CD45RA^–^ T cells/kg)	Lymphocyte count before infusion (×10^3^/μl)	Best lymphocyte count after infusion (×10^3^/μl)	Viral copies or GMN before infusion (copies/μl)	Best response after infusion (copies/μl)	Best T cell donor chimerism (%)	Concomitant therapy during infusions	Duration of concomitant therapy during infusion	Outcome
1	14	10 × 10^7^/kg	0.53	1.12	7.5 × 10^3^	<1,000	0.5-0.25	IS withdrawn. IGs	2 months	Alive with stabilization of renal function
2	12	5.01 × 10^6^/kg	0.38	1.44	2.3 × 10^3^	0	Not performed	Ganciclovir/Valganciclovir then replaced by Letermovir. Specific HCMV IGs (parenteral and intrathecal).	Ganciclovir/Valganciclovir (6 months). Letermovir. Specific HCMV IGs (6 months).	Alive with encephalitis sequelae
3	6	5.4 × 10^5^/kg	0.9	1.46	<1,000	0	Not detected	Letermovir and weekly IGs	4 months	Died of septic shock (not associated with therapy)
4	11	1.2 × 10^5^/kg 5 × 10^5^/kg 1 × 10^6^/kg	0.89	1.03	0.52	Serum and BAL negative GMN	Not detected	Posaconazole	1 year	Alive and Lansky score 100%
5	10	1.53 × 10^7^/kg	0.02	0.54	1.07 × 10^5^	<3,500	1	Chemotherapy. IS withdrawn. IGs	1 month	Alive in CR after EBV CTLs
6	6	1 × 10^7^/kg	0.04	0.18	0	0	1, <1	Rituximab. Third line chemotherapy. IGs every 2 weeks	1 month	Alive in CR after MUD HSCT

In cases of leukemia, established doses of 1 × 10^6^ escalating to 1 × 10^7^ cells/kg are infused. After transplantation, the doses established for CTLs infusions are very variable, from 1 × 10^3^ to 1 × 10^9^ cells/kg (21). However, beyond the transplantation setting, there are no established doses for these procedures, but there are a number of examples that have proven to be safe (20). In this study, when the patients were at risk of GvHD, the minimum dose infused was 1 × 10^5^ cells/kg of weight; however, the normal dose was usually 1 × 10^6^ cells/kg. In all cases, subsequent doses were decided based on the clinical evaluation, polymerase chain reaction (PCR) levels and immunologic follow-up.

### 2.5. Lymphocyte reconstitution, viral load and microchimerism

Lymphocytes levels were measured by a routine hemogram, and viral load was defined by PCR. Microchimerism analysis was performed in the Community of Madrid Transfusion Center (Madrid, Spain) as previously described (20). For each donor-recipient pair, DNA was isolated using the QIAamp Blood Kit (Qiagen, Germany). The microchimerism analysis was monitored weekly for 3-4 consecutive weeks, and performed based on detecting insertion/deletion polymorphism (INDELs) by qPCR technology (sensitivity 0.01–0.05%). To this end, commercial reagents for screening of informative alleles (Mentype DIPscreen, Biotype, Dresden, Germany) and quantitative chimerism analysis (Mentype DIPquant qPCR, Biotype) were employed. The percentage of donor alleles was calculated based on the DDCt qPCR method using Chimerism Monitor 2.1 software (Biotype), with b-globin as the reference gene.

## 3. Results

### 3.1. Detection of pathogen-specific CD45RA^–^ memory T-cells

**FIGURE 2:**
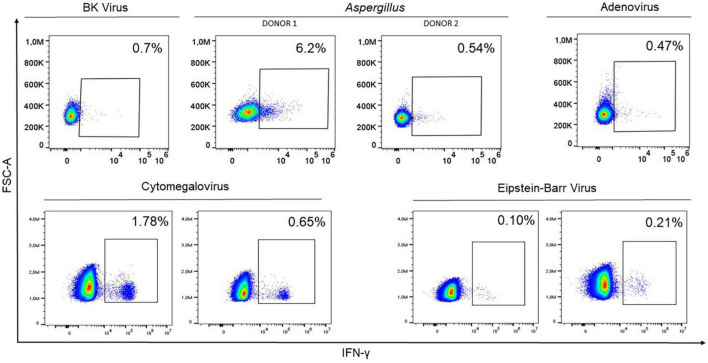
Representation of interferon-gamma (IFN-γ) expression by flow cytometry in 8 potential donors within the CD45RA^–^ subpopulation after co-culturing peripheral blood mononuclear cells with the mixture of 4 BKV peptides (ST, LT, VP1, VP2), *Aspergillus fumigatus* Lysate at a final concentration of 1 μg/ml for *Aspergillus*; PepTivator^®^ AdV Select for Adenovirus; PepTivator^®^ CMV pp65 for HCMV; and EBV-PT consensus premium grade human for EBV. In all cases, the cell response was sufficient for use in cell therapy. Donor apheresis was therefore performed, and CD45RA^–^ T cells were isolated and infused.

Potential donors for adoptive cell therapy were tested to check their response against different pathogens, namely HCMV, AdV, EBV, BKV, and *Aspergillus*. The presence of CD45RA^–^ memory T cells responsive to all pathogens was detected in all donors (Figure 2). The donors therefore underwent apheresis and subsequent infusions to patients were performed. However, the infection was resolving in patient with AdV; thus, cells were stored for future use. This detection process is effective and could be an accepted and extended diagnostic procedure in the near future.

### 3.2. Adoptive cell therapy with CD45RA^–^ cells containing pathogen-specific memory T-cells to treat infectious diseases

Four of the six patients included in this study had severe refractory infections that where not responding to classical therapies. An adoptive cell therapy with CD45RA^–^ T cells containing pathogen-specific memory T cells therefore appeared as the best treatment option. The four cases are described below. Table 3 presents a summary of the response and treatment outcomes with familial CD45RA^–^ T cells.

#### 3.2.1. BKV nephritis (patient 1)

The first patient was a 37-year-old woman who underwent a kidney transplantation due to chronic kidney failure secondary to focal and segmental glomerulonephritis. The patient was prescribed rejection prophylaxis (prednisone, tacrolimus and everolimus), and as a result she developed lymphopenia, with lymphocytes counts of 670/μl. Approximately 9 months after transplantation, and despite changing the medication, the patient showed a progressive worsening of the renal function and a rise of both creatinine levels and plasmatic BKV copies. The patient was therefore diagnosed with BKV nephritis.

**FIGURE 3:**
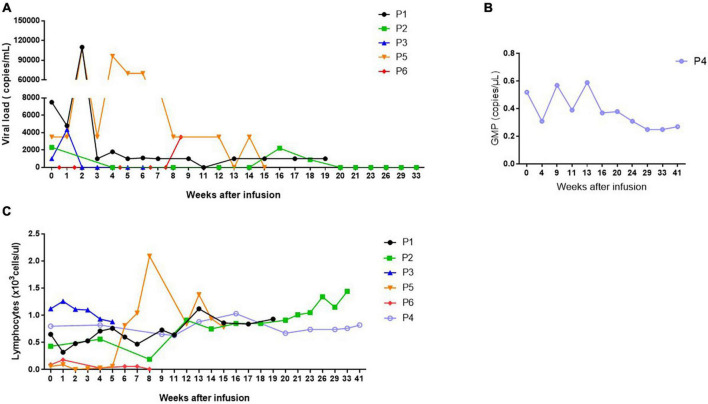
Graphical representation of **(A)** viral load (copies/ml), **(B)** galactomannan (GMP) (copies/μl) and **(C)** lymphocyte (×10^3^ cells/μl) progression while the infusions were performed. As expected, the viral load and GMP decrease with adoptive cell therapy with CD45RA^–^ T-cell infusions containing pathogen-specific memory T cells, while lymphocyte count increase.

Given the situation, and after an ineffective immunosuppression-therapy reduction, Leflunomide plus intravenous immunoglobulins (IGs) were suggested. For 4 months the patient was not improving, so cell therapy in combination with standard therapy was suggested. One of the patients’ brother had identical HLA and 0.4% of CD3^+^ cells that were IFN-γ^+^ (Table 2 and Figure 2). Thus, weekly infusions of 1 × 10^7^ cells/kg of CD45RA^–^ T donors’ cells started. A week after first infusion, T-cell donor chimerism was 0.5% and after the second infusion it was 0.25%. Then it was undetectable. A month later, the patient showed stable kidney function and viral replication dropped to the minimum. After 2 months, lymphocyte counts increased (Figure 3); therefore IGs were suspended and infusions were performed every 2 weeks for 4 months and then monthly until the present. The patient is currently undergoing monthly infusions, and there are no signs of worsening renal function while the viral replication continued under control, with rising lymphocytes levels. The infusion of CD45RA^–^ T-cells showed no side effects, and there was no GvHD. The total duration of the treatment remains uncertain.

#### 3.2.2. HCMV encephalitis (patient 2)

Patient 2, a 19-year-old man with chronic granulomatous disease who underwent HSCT from a haploidentical match unrelated donor, was receiving DLIs every 2 weeks to promote immune reconstitution in combination with GvHD prophylaxis (methylprednisolone and cyclosporine). Two months after HSCT patient suffer a HCMV reactivation, treated with valganciclovir, and 2 months later, the patient developed an encephalitis caused by HCMV with high viral copies in blood and cerebrospinal fluid (CSF). At this point the immune reconstitution was incomplete and his total lymphocyte count was 50/μl.

To treat HCMV encephalitis correctly, and since valganciclovir was not effective, patient’s sensitivity to antivirals was tested and he was sensitive to foscarnet, ganciclovir and cidofovir. Then, patient received foscarnet and ganciclovir first, suspended because of their high toxicity, followed by IGs weekly. After 2 weeks ganciclovir was reintroduced, without side-effects this time, but without any improve of the patient’s state. After 2 months with a progressive worsening and with elevated viral copies in blood and CSF, adoptive cell therapy with CD45RA^–^ T-cells was indicated. The patient’s brother shared three HLA alleles and a total of 1.61% of CD3^+^ cells that were IFN-γ^+^ against HCMV (Table 2 and Figure 2). Infusions of 5 × 10^6^ cells/kg of CD45RA^–^ cells were combined with ganciclovir and IGs. Only 2 weeks after infusion the patient showed progressive neurological improvement of his symptoms and a decrease of HCMV viral copies in CSF. Blood viral copies were undetectable 1 month after the infusion and CSF viral copies were undetectable 2 months later. The number of lymphocytes progressively increased and stood at 1,000/μl seven months later (Figure 3). Microchimerism was not performed in this case.

To date, the patient has been clinically stable without new neurologic symptoms or reactivation, but dealing with important sequelae due to HCMV ventricle-encephalitis. He maintains treatment with letermovir and monthly infusions of CD45RA^–^ T cells to accelerate immune reconstitution. The CD45RA^–^ T-cells infusions were safe and effective in this patient, showing no side effects or GvHD and with a decrease of viral load and an increase in lymphocyte number.

#### 3.2.3. HCMV reactivation (patient 3)

The next patient was a 7-year-old boy with Trichohepatoenteric Syndrome, who underwent multivisceral transplantation with spleen preservation at 6 years of age. The patient developed GvHD with skin and eye involvement 10 months post-transplantation due to the intestinal graft resident lymphocytes. He therefore started on methylprednisolone as immunosuppression therapy, and rejection and HCMV prophylaxis (sirolimus and valganciclovir respectively). However, virus reactivation was detected and he started on treatment with ganciclovir and intravenous IGs, reduced immunosuppression dosage and, as the patient’s GvHD was also worsening, he initiated treatment with ruxolitinib, resulting in a decreasing lymphocyte count.

After one month, and despite changing medication several times (foscarnet for one month and then ganciclovir for another month), new rise in the viral copies was detected. Patient’s mother was HLA-haploidentical and has 0.62% of CD3^+^ cells that were IFN-γ^+^ (Table 2 and Figure 2). Thus, the patient started weekly infusions of donor’s CD45RA^–^ T cells at a dose of 5 × 10^5^ cells/kg. After two infusions, the viral copies were undetectable and remained so throughout the treatment (Figure 3), although microchimerism was not detected.

A total of 6 infusions were administered, with no side effects and controlling HCMV infection. However, lymphocyte count did not improve and GvHD was worsening despite an immunosuppression increase. The infusions were therefore suspended in case they were contributing to the worsening of GvHD, even if this was not suspected since we were not able to detect even microchimerism from the mother (all the chimerism detected was from the intestinal graft donor). Nevertheless, his clinical condition continued progressively worsening until he finally died of septic shock.

#### 3.2.4. Lung disseminated invasive aspergillosis (patient 4)

The last patient was a 15-year-old girl with severe combined immunodeficiency, specifically a cytotoxic T-lymphocyte antigen 4 (CTLA-4) haplo-insufficiency. She underwent HSCT from an identical match unrelated donor and, despite being under fungal prophylaxis with micafungin, a high level of galactomannan (GMN) was detected 8 months after the HSCT. She initiated treatment with voriconazole and 4 months later, it was changed by amphotericin B because of an azole resistance. Infection was in that way controlled.

However, as an HSCT complication, the patient developed steroid-refractory bronchiolitis obliterans syndrome 7 months later, which required immunosuppression therapy with abatacept. As a result, GMN levels rose again. In a PET-CT scan invasive aspergillosis with some lung aspergillomas were detected. The treatment, consisting in surgery and azoles, controlled the infection. Same events happened one year later, however, this time GMN levels remained high, despite the new aspergilloma-removing surgery and the treatment with voriconazole and with amphotericin B. A schematic representation of the evolution of the patient is represented in Supplementary Figure 1. At this point, the HSCT donor was studied and was found immunologically competent against *Aspergillus* (Donor 1, Figure 2), but was not available for apheresis. Therefore, patient’s father, who was HLA-haploidentical and has 0.28% of CD3^+^ cells that were IFN-γ^+^ was chosen as a donor (Donor 2, Figure 2). Patient get monthly infusions of 1 × 10^5^ cells/kg CD45RA^–^ T-cells combined with posaconazole. Two months later there was a significant reduction in GMN levels but with a few rebounds (Figure 3), so the dose was increased to 5 × 10^5^/kg and the infusions performed every 2 weeks.

Although in this case, we saw no significant changes in lymphocyte counts (Figure 3) and no microchimerism was found, GMN levels remained negative 4 months after the therapy, so the infusions were changed to monthly with a higher dose of 1 × 10^6^/kg. The patient maintains this therapy and is currently clinically stable with no new rebounds in GMN levels more than one year and a half after cell therapy start. No side effects were found associated with the cell infusions, there were no signs of GvHD and we observed an improvement in the lymphocyte recovery.

### 3.3. Adoptive cell therapy with CD45RA^–^ T cells containing EBV-specific memory T cells against EBV associated lymphoproliferative syndromes

Two of the six patients involved in our study experienced PTLD associated to EBV and were not responding to standard treatments. The patients therefore required cellular therapy to control the viral reactivation. The two cases are described below. Table 3 provides a summary of the response and treatment outcomes with familial CD45RA^–^ T-cells.

#### 3.3.1. PTLD after a multivisceral transplantation (patient 5)

This patient is a 12-year-old girl with intestinal failure secondary to postsurgical superior mesenteric vein thrombosis, who underwent multivisceral transplantation. She developed acute skin GvHD three months after the transplantation and was treated with high doses of corticosteroids, ruxolitinib and photopheresis for a month. After that, the patient get rejection prophylaxis; consequently, she developed severe lymphopenia, with a total lymphocyte count of 230/μl. At that moment, a blood reactivation of EBV was detected, so immunosuppression was minimized and rituximab was initiated. After a while, the patient developed a monomorphic PTLD, a diffuse large B-cell lymphoma (DLBCL). A PET-CT showed supra-diaphragmatic (cervical, axillary, and pleural effusion) and infra-diaphragmatic (liver injury and doubtful renal) involvement (stage III according to Murphy’s classification).

Immunosuppression therapy was minimized, rituximab was maintained and the patient started chemotherapy following Inter-B-NHL ritux 2010 protocol in the high-risk group B. She received one COP cycle and two R-COPADM. There was an initial good response of the DLBCL to chemotherapy, but viral copies of EBV remained elevated in serum despite rituximab, and the patients’ lymphocytes count was still very low.

In this context, patient’s father was studied as potential donor. He presented a 0.17% of CD3^+^ IFN-γ^+^ cells and he was HLA-haploidentical, so the patient initiated infusions of 1.53 × 10^7^ cells/kg of CD45RA^–^ T-cells twice a week. A 1% of T-cell microchimerism was detected after the first two infusions and it get undetectable after that. EBV copies initially decreased, but did not go negative, and the patient still has lymphopenia (Figure 3). No side effects or GvHD were observed after infusions.

After 3 months, the patient had a lymphoma in progression in spite of first-line chemotherapy, and an EBV reactivation without significant response to rituximab and familial CD45RA^–^ T-cells. For this reason, chemotherapy and CD45RA^–^ T- cells infusions were suspended, and infusions of specific CTLs against EBV twice a week started. After two infusions of 5.7 × 10^7^ cells/ml of specific CTLs, the number of viral copies decreased significantly, lymphocyte counts improved considerably and the patient showed good clinical response. After 6 infusions, EBV copies in blood were undetectable, the patient presented normal lymphocyte counts and a partial response of PTLD was finally achieved. The treatment with specific CTLs was then interrupted. To date, 14 months after the end of treatment, the patient is still clinically stable without signs of disease progression. No side effects related to the use of CD45RA^–^ T-cells or CTLs were observed.

#### 3.3.2. Diffuse large B-cell lymphoma and EBV infection in a patient with primary immunodeficiency (patient 6)

The last patient was a 9-year-old girl with a diagnosis of unaffiliated primary congenital immunodeficiency. She had a history of primary EBV infection when she was 5 years old, and since then she maintained persistently positive viral copies in serum.

At 8 years of age, the patient was diagnosed with a DLBCL (stage III according to Murphy’s classification). She started standard therapy with rituximab and first line chemotherapy (prednisone and cyclophosphamide). Three months later she started second line therapy following Inter-B-NHL protocol as high-risk group C1, receiving 2 COPADM cycles and one CYVE. Finally, 2 months later she received a third line of treatment with a first R-ICE cycle as rescue therapy. After rescue therapy, EBV copies became negative, but infusions of CD45RA^–^ T-cells were performed in order to prevent future reactivations. Her mother was HLA-haploidentical and presented 0.3% of CD3^+^ IFN-γ^+^ T-cells (Table 2 and Figure 2). First, weekly infusions of 1 × 10^7^ CD45RA^–^ cells/kg were performed, without any side effects or GvHD. No microchimerism was detected. A second R-ICE was administered during the infusions, and no significant changes in the total lymphocyte count was observed, probably due to concomitant treatment with chemotherapy. The patient received a total of 6 infusions with no EBV reactivations, which allow HSCT. More than one year after the HSCT, the patient is still at complete remission, with no new EBV reactivation.

## 4. Discussion

Here we present a treatment focused on the ability to clear various viruses (HCMV, AdV, BKV, EBV), and the fungi *Aspergillus* by mDLI of CD45RA^–^ cells containing pathogen-specific memory T-cells of a familial donor. We conducted a study with six patients, children and adults who were immunosuppressed and who experienced either an infection or reactivation of different viruses or EBV-associated lymphoproliferative disease. Given that these patients did not respond to classical treatments, this therapy provides a new, safe and effective curative procedure. These mDLIs were used as well in a preventive way to avoid reactivations, as in patient 6. We present as well the methodology to detect, isolate and infuse CD45RA^–^ T cells in this novel cell therapy.

Cell reconstitution therapies are routinely performed to recover the immune system after HSCT (5–7, 22), a process that is essential for patient survival. Cells from the HSCT donor are usually employed to perform this recovery, but if the donor does not have memory cells against a specific pathogen, the patient is at risk of infection or reactivation of a latent virus. Thereby, cell therapy with CD45RA^–^ T cells appears to be the best option for avoiding and controlling infections after HSCT. What is more, in immunocompromised patients who have the same disease and progress with lymphopenia or lack of memory cells against a specific virus, CD45RA^–^ T cells are a life drug alternative to conventional treatments. In many cases, there are more effective or are the only option when there is a lack of appropriate drugs. Indeed, more and more resistances to anti-viral or anti-fungal drugs urge to develop new solutions and treatments for not only extreme but also common infections in healthy donors without comorbidities or previous pathologies (23, 24).

Adoptive cell therapy with CD45RA^–^ T cells is as well an alternative to CTLs, usually used to treat infections in immunocompromised patients. Some examples employed to treat the infections described in this article are the clinical trial by Muftuoglu et al., for treating multifocal leukoencephalopathy caused by the John Cunningham Virus (genetically similar to BKV) (10); or the use of CTLs proposed by Bao et al., to treat HCMV infections in stem cell transplantation patients (25). However, CTLs need to be isolated, characterized and expanded *in vitro*, a costly and lengthy process that our therapy is not subject to. The therapy that we propose is not considered an advanced cell therapy and therefore does not require substantial cell modification, making the process easier and cheaper, without losing effectiveness.

One essential factor to consider in our therapy is the presence of a repertoire of memory lymphocytes with proven antiviral activity in the donor’s CD45RA^–^ subpopulation. To confirm that activity we used the IFN-γ production in presence of small peptides or a lysate that simulate the contact with a real pathogen. Previous studies by our laboratory reported the presence of SARS-CoV-2-specific T-cell subpopulations within CD45RA^–^ memory T cells in the blood of convalescent donors that infused might clear virally infected cells and confer T-cell immunity for subsequent reinfections, representing an off-the-shelf living drug (19, 20). In this study, we are applying the same technology to treat other infectious diseases, with visible results.

The other essential factor to take in account when selecting the best donor is the HLA compatibility, which must be at least partial to permit TCR/HLA pathogen recognition. CD45RA^–^ T cells are mainly CD4^+^ cells, with a less proportion of CD8^+^ cells. In the case of HCMV infections, it is known that deficiencies in the response of class I HLA-restricted CD8^+^ cytotoxic T lymphocytes are important in the pathogenesis of the disease in immunocompromised recipients of allogeneic transplants (26). However, in the two HCMV patients that were infused in this study, a clearance of the virus was achieved. Therefore, we can obtain a good response despite the less proportion of CD8^+^ cells in our product. Moving on, EBV presentation is mediated by HLA-class II alleles before the latent phase of the infection; CD4^+^ T cells recognize particles presented by MHC-class II in the surface of infected B lymphocytes and eliminate them. In later stages of the infection, HLA class I alleles are the ones that induce a strong specific cytotoxic CD8 + T cell response (27). Therefore, CD45RA^–^ T cells might be more effective in early stages of the infection or when there is not an active infection. On the contrary, CTLs infusions would be more effective when the infection is spread, as we observed in our study patients. Indeed, patient 5 needed CTLs infusions to contain the infection, while in patient 6, who was receiving the infusions in a prophylactic manner, the CD45RA^–^ T-cells were effective avoiding viral load increase. It is true that CTLs are more effective, but they are also more alloreactive, what increases the risk of having GvHD or tissue inflammation. On the other side, BKV immunity is associated with a multifunctional population of CD4 + T cells with both T-helper and T-cytotoxic properties (28). That might be the reason why CD45RA^–^ T cells infusion in patient 1 was completely effective. Finally, immunology of *Aspergillus* is complex and not completely defined, but T regulatory cells (which were contained in CD45RA^–^ T-cells infused to the patient 4) might have a crucial role in the responses against this pathogen (29, 30).

In this study we performed weekly and monthly mDLI of CD45RA^–^ T cells to patients who had HCMV, BKV, EBV, and *Aspergillus* infections that progressed with lymphopenia and who did not respond to standard therapies. We observed improvement in the general symptoms for a number of the patients, as well as increased lymphocyte counts and reduced viral and GMP loads, sometimes to zero. When performed, microchimerism between donors and patients cells was observed after the first and second infusion, and then it was undetectable, coinciding with the reduction of viral loads and the increase of lymphocyte counts. Therefore, when autologous lymphocytes get recovered, donor’s lymphocytes are reduced to minimum and are not detected. None of the patients experienced adverse effects due to infusions or GvHD reactions, and the infusions did not interfere with the treatments the patients were undergoing for other diseases. Thereby, this therapy can be combined with other procedures like HSCT, CTLs infusions, and different antiviral and antifungal drugs.

However, this study has some limitations. A larger patient cohort would be useful for studying specific reactions and characteristics of the infections. Obtaining more information and follow up of the patients would be of use; for example, more data on microchimerism, or donor’s CD45RA^–^ T cells function and interactions in the patient is essential for future research. Also a study of how immune lymphocyte reconstitution develops and a phenotyping of the different immune system cells subsets would be very interesting for future approaches Finally, as we propose, the possibility of generating a biobank of living drugs accessible to all patients will be useful for providing this treatment to those who might need it.

In conclusion, after this study we can affirm that the use of familial multiple CD45RA^–^ T cells containing specific memory T-cells of a third-party donor is a feasible, safe and potential effective approach for treating severe pathogenic diseases in immunocompromised patients non-responders to antiviral treatments or with side effects due to their toxicity. This therapy provides and improves the cellular immunity of the patient until he recovers its own one and protects from opportunistic infections. Furthermore, this approach might be of universal use with fewer institutional and regulatory barriers, since Good Manufacturing Practices are not required.

## Data availability statement

The original contributions presented in this study are included in the article/Supplementary material, further inquiries can be directed to the corresponding author.

## Ethics statement

Ethical review and approval was not required for the study on human participants in accordance with the local legislation and institutional requirements. Written informed consent to participate in this study was provided by the participants’ legal guardian/next of kin. Written informed consent was obtained from the individual(s), and minor(s)’ legal guardian/next of kin, for the publication of any potentially identifiable images or data included in this manuscript.

## Author contributions

AP-M: conceptualization and resources. KAS and AP-M: methodology. KAS, CE, and AP-M: research and writing of the original draft. AP-M and CF: supervision. All authors: writing, reviewing, and editing.

## References

[1] Thorley-Lawson D Gross A. Persistence of the Epstein–Barr virus and the origins of associated lymphomas. *N Engl J Med.* (2004) 350:1328–37. 10.1056/NEJMra032015 15044644

[2] Waller EC Day E Sissons JG Wills MR. Dynamics of T cell memory in human cytomegalovirus infection. *Med Microbiol Immunol.* (2008) 197:83–96. 10.1007/s00430-008-0082-5 18301918

[3] Weist BJD Schmueck M Fuehrer H Sattler A Reinke P Babel N. The role of CD4+ T cells in BKV-specific T cell immunity. *Med Microbiol Immunol.* (2014) 203:395–408. 10.1007/s00430-014-0348-z 25052009

[4] Leen A Heslop H Brenner M. Antiviral T-cell therapy. *Immunol Rev.* (2014) 258:12–29. 10.1111/imr.12138 24517423 PMC3927231

[5] Wang L Janes M Kumbhojkar N Kapate N Clegg J Prakash S Cell therapies in the clinic. *Bioeng Transl Med.* (2021) 6:e10214. 10.1002/btm2.10214 PMC8126820 34027097

[6] Gasior M Ferreras C de Paz R Bueno D Mozo Y Sisinni L The role of early natural killer cell adoptive infusion before engraftment in protecting against human herpesvirus-6B encephalitis after naïve T-cell-depleted allogeneic stem cell transplantation. *Transfusion.* (2021) 61:1505–17. 10.1111/trf.16354 33713461

[7] Triplett B Shook D Eldridge P Li Y Kang G Dallas M Rapid memory T-cell reconstitution recapitulating CD45RA-depleted haploidentical transplant graft content in patients with hematologic malignancies. Bone Marrow Transplant. 2015 Jul;50(7):968-77. Erratum in. *Bone Marrow Transplant.* (2015) 50:1012. 10.1038/bmt.2014.324 25665048 PMC4636007

[8] Maschan M Blagov S Shelikhova L Shekhovtsova Z Balashov D Starichkova J Low-dose donor memory T-cell infusion after TCR alpha/beta depleted unrelated and haploidentical transplantation: results of a pilot trial. *Bone Marrow Transplant.* (2018) 53:264–73. 10.1038/s41409-017-0035-y 29269793

[9] Brodszki N Turkiewicz D Toporski J Truedsson L Dykes J. Novel treatment of severe combined immunodeficiency utilizing ex-vivo T-cell depleted haploidentical hematopoietic stem cell transplantation and CD45RA+ depleted donor lymphocyte infusions. *Orphanet J Rare Dis.* (2016) 11:5. 10.1186/s13023-016-0385-3 26768987 PMC4714422

[10] Muftuoglu M Olson A Marin D Ahmed S Mulanovich V Tummala S Allogeneic BK virus-specific T cells for progressive multifocal leukoencephalopathy. *N Engl J Med.* (2018) 379:1443–51. 30304652 10.1056/NEJMoa1801540 PMC6283403

[11] Gerdemann U Katari UL Papadopoulou A Keirnan JM Craddock JA Liu H Safety and clinical efficacy of rapidly-generated trivirus-directed T cells as treatment for adenovirus, EBV, and CMV infections after allogeneic hematopoietic stem cell transplant. *Mol Ther.* (2013) 21:2113–21. 10.1038/mt.2013.151 23783429 PMC3831033

[12] Withers B Blyth E Clancy L Yong A Fraser C Burgess J Long-term control of recurrent or refractory viral infections after allogeneic HSCT with third-party virus-specific T cells. *Blood Adv.* (2017) 1:2193–205. 10.1182/bloodadvances.2017010223 29296867 PMC5737128

[13] Neuenhahn M Albrecht J Odendahl M Schlott F Dössinger G Schiemann M Transfer of minimally manipulated CMV-specific T cells from stem cell or third-party donors to treat CMV infection after allo-HSCT. *Leukemia.* (2017) 31:2161–71. 10.1038/leu.2017.16 28090089

[14] Kaeuferle T Krauss R Blaeschke F Willier S Feuchtinger T. Strategies of adoptive T -cell transfer to treat refractory viral infections post allogeneic stem cell transplantation. *J Hematol Oncol.* (2019) 12:13. 10.1186/s13045-019-0701-1 30728058 PMC6364410

[15] Feuchtinger T Opherk K Bethge W Topp M Schuster F Weissinger E Adoptive transfer of pp65-specific T cells for the treatment of chemorefractory cytomegalovirus disease or reactivation after haploidentical and matched unrelated stem cell transplantation. *Blood.* (2010) 116:4360–7. 10.1182/blood-2010-01-262089 20625005

[16] Bremm M Krastel T Cappel C Zimmermann O Pfeffermann L Katzki V Depletion of CD45RA+ T cells: advantages and disadvantages of different purification methods. *J Immunol Methods.* (2021) 492:112960. 10.1016/j.jim.2021.112960 33417916

[17] Triplett BM Muller B Kang G Li Y Cross SJ Moen J Selective T-cell depletion targeting CD45RA reduces viremia and enhances early T-cell recovery compared with CD3-targeted T-cell depletion. *Transpl Infect Dis*. (2018) 20:e12823. 10.1111/tid.12823 29178554 PMC5809307

[18] Müller N Landwehr K Langeveld K Stenzel J Pouwels W van der Hoorn M Generation of alloreactivity-reduced donor lymphocyte products retaining memory function by fully automatic depletion of CD45RA-positive cells. *Cytotherapy.* (2018) 20:532–42. 10.1016/j.jcyt.2018.01.006 29500069

[19] Ferreras C Pascual-Miguel B Mestre-Durán C Navarro-Zapata A Clares-Villa L Martín-Cortázar C SARS-CoV-2-specific memory T lymphocytes from COVID-19 convalescent donors: identification, biobanking, and large-scale production for adoptive cell therapy. *Front Cell Dev Biol.* (2021) 9:620730. 10.3389/fcell.2021.620730 33718360 PMC7947351

[20] Pérez-Martínez A Mora-Rillo M Ferreras C Guerra-García P Pascual-Miguel B Mestre-Durán C Phase I dose-escalation single centre clinical trial to evaluate the safety of infusion of memory T cells as adoptive therapy in COVID-19 (RELEASE). *Eclinicalmedicine.* (2021) 39:101086. 10.1016/j.eclinm.2021.101086 34405140 PMC8361305

[21] Pei X Zhao X Liu X Mo X Lv M Xu L Adoptive therapy with cytomegalovirus-specific T cells for cytomegalovirus infection after haploidentical stem cell transplantation and factors affecting efficacy. *Am J Hematol.* (2022) 97:762–9. 10.1002/ajh.26535 35293011

[22] Ogonek J Kralj Juric M Ghimire S Varanasi P Holler E Greinix H Immune reconstitution after allogeneic hematopoietic stem cell transplantation. *Front Immunol.* (2016) 7:507. 10.3389/fimmu.2016.00507 27909435 PMC5112259

[23] Strasfeld L Chou S. Antiviral drug resistance: mechanisms and clinical implications. *Infect Dis Clin.* (2010) 24:809–33. 10.1016/j.idc.2010.07.001 20674805

[24] Fisher MC Alastruey-Izquierdo A Berman J Bicanic T Bignell EM Bowyer P Tackling the emerging threat of antifungal resistance to human health. *Nat Rev Microbiol.* (2022) 20:557–71. 35352028 10.1038/s41579-022-00720-1 PMC8962932

[25] Bao L Cowan M Dunham K Horn B McGuirk J Gilman A Adoptive immunotherapy with CMV-specific cytotoxic T lymphocytes for stem cell transplant patients with refractory CMV infections. *J Immunother.* (2012) 35:293–8. 22421947 10.1097/CJI.0b013e31824300a2 PMC3306600

[26] Walter E Greenberg P Gilbert M Finch R Watanabe K Thomas E Reconstitution of cellular immunity against cytomegalovirus in recipients of allogeneic bone marrow by transfer of T-cell clones from the donor. *N Engl J Med.* (1995) 333:1038–44. 10.1056/NEJM199510193331603 7675046

[27] Mautner J Bornkamm GW. The role of virus-specific CD4+ T cells in the control of Epstein-Barr virus infection. *Eur J Cell Biol.* (2012) 91:31–5. 21458882 10.1016/j.ejcb.2011.01.007

[28] Ramaswami B Popescu I Macedo C Luo C Shapiro R Metes D The polyomavirus BK large T-antigen-derived peptide elicits an HLA-DR promiscuous and polyfunctional CD4+ T-cell response. *Clin Vaccine Immunol.* (2011) 18:815–24. 10.1128/CVI.00487-10 21367979 PMC3122532

[29] Bacher P Kniemeyer O Teutschbein J Thön M Vödisch M Scheffold A Identification of immunogenic antigens from Aspergillus fumigatus by direct multiparameter characterization of specific conventional and regulatory CD4+ T cells. *J Immunol.* (2014) 193:3332–43. 10.4049/jimmunol.1400776 25172488

[30] Rivera A Van Epps HL Hohl TM Rizzuto G Pamer EG. Distinct CD4+-T-cell responses to live and heat-inactivated Aspergillus fumigatus conidia. *Infect immun.* (2005) 73:7170–9. 10.1128/IAI.73.11.7170-7179.2005 16239511 PMC1273880

