# Supplementary figures and images for: Familial CD45RA– T cells to treat severe refractory infections in immunocompromised patients
Source: Front Med (Lausanne). 2023 Feb 8;10:1083215. doi: 10.3389/fmed.2023.1083215 (PMC9944023; doi:10.3389/fmed.2023.1083215)

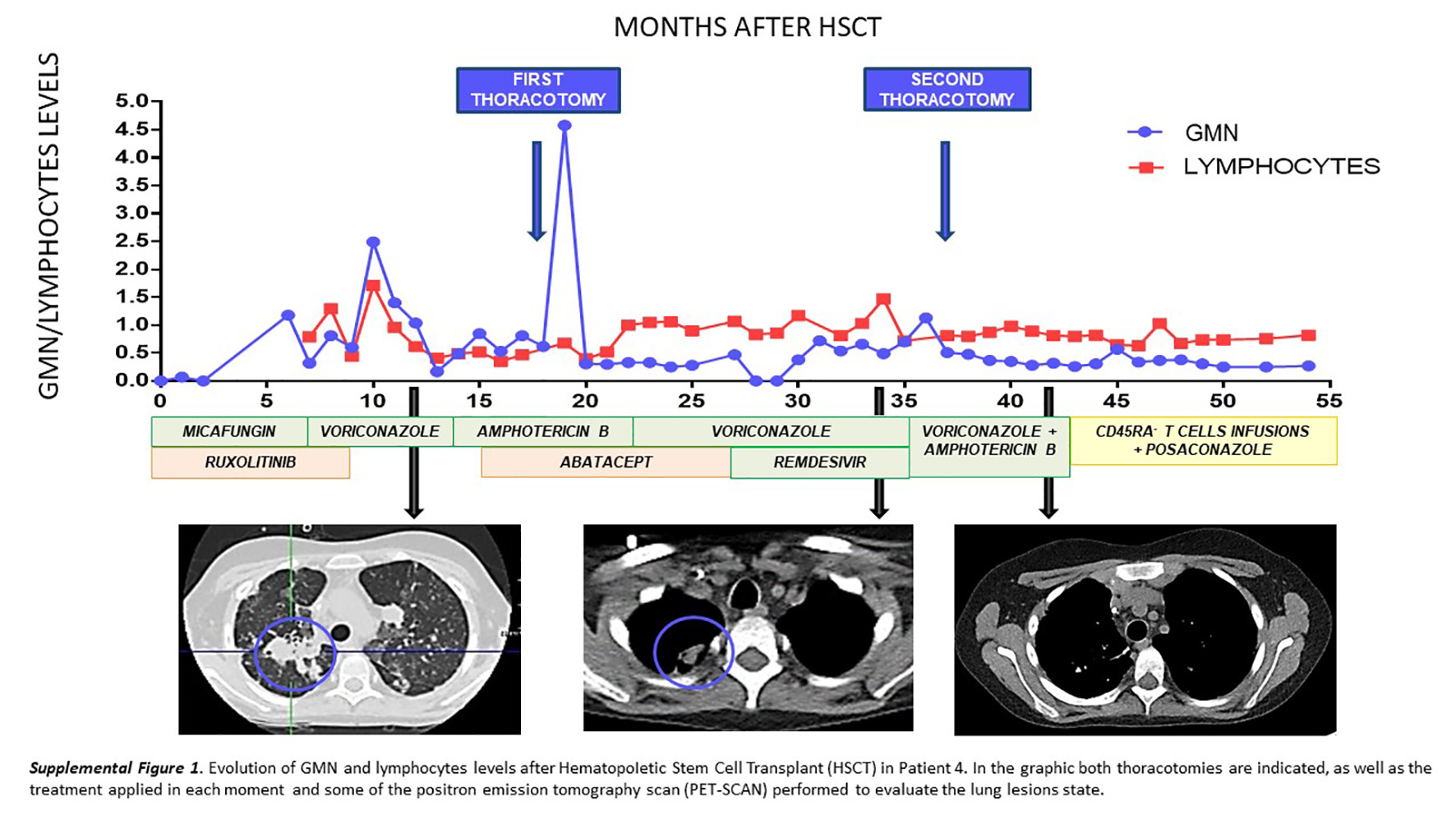

Supplement: Supplementary file 1 [file Image_1.jpeg]
